# The Sorghum (*Sorghum bicolor*) *Brown Midrib 30* Gene Encodes a Chalcone Isomerase Required for Cell Wall Lignification

**DOI:** 10.3389/fpls.2021.732307

**Published:** 2021-12-02

**Authors:** Hannah M. Tetreault, Tammy Gries, Sarah Liu, John Toy, Zhanguo Xin, Wilfred Vermerris, John Ralph, Deanna L. Funnell-Harris, Scott E. Sattler

**Affiliations:** ^1^Wheat, Sorghum and Forage Research Unit, Agricultural Research Service, United States Department of Agriculture, Lincoln, NE, United States; ^2^Department of Agronomy and Horticulture, University of Nebraska–Lincoln, Lincoln, NE, United States; ^3^Department of Biochemistry, The DOE Great Lakes Bioenergy Research Center, Wisconsin Energy Institute, University of Wisconsin, Madison, WI, United States; ^4^Plant Stress and Germplasm Development Unit, Agricultural Research Service, United States Department of Agriculture, Lubbock, TX, United States; ^5^Department of Microbiology and Cell Science, UF Genetics Institute, University of Florida, Gainesville, FL, United States; ^6^Department of Plant Pathology, University of Nebraska–Lincoln, Lincoln, NE, United States

**Keywords:** lignin biosynthesis, flavonoid biosynthesis, tricin lignin, mutant, NMR, monolignol

## Abstract

In sorghum (*Sorghum bicolor*) and other C_4_ grasses, *brown midrib* (*bmr*) mutants have long been associated with plants impaired in their ability to synthesize lignin. The *brown midrib 30* (*Bmr30*) gene, identified using a bulk segregant analysis and next-generation sequencing, was determined to encode a chalcone isomerase (CHI). Two independent mutations within this gene confirmed that loss of its function was responsible for the brown leaf midrib phenotype and reduced lignin concentration. Loss of the *Bmr30* gene function, as shown by histochemical staining of leaf midrib and stalk sections, resulted in altered cell wall composition. In the *bmr30* mutants, CHI activity was drastically reduced, and the accumulation of total flavonoids and total anthocyanins was impaired, which is consistent with its function in flavonoid biosynthesis. The level of the flavone lignin monomer tricin was reduced 20-fold in the stem relative to wild type, and to undetectable levels in the leaf tissue of the mutants. The *bmr30* mutant, therefore, harbors a mutation in a phenylpropanoid biosynthetic gene that is key to the interconnection between flavonoids and monolignols, both of which are utilized for lignin synthesis in the grasses.

## Introduction

*Sorghum bicolor* is an economically important C_4_ grass, the fifth most important cereal crop in the world, which is grown as a grain, forage, sugar, and lignocellulosic bioenergy crop. Although sorghum can grow in a wide range of environments, its production is usually associated with hot and dry regions because of its high water-use efficiency and drought tolerance. Sorghum is, therefore, being developed as a potential bioenergy crop due to its ability to grow under sub-optimal conditions and climate resiliency (van der Weijde et al., 2013). Utilizing plant cell wall biomass as a renewable resource for the production of energy and fuels has become a major research focus (Vermerris et al., 2007). Plant cell walls predominantly consist of the polysaccharides cellulose and hemicelluloses along with the phenolic polymer lignin. Lignin is polymerized through radical coupling processes in which hydroxycinnamyl alcohols, mainly *p*-coumaryl, coniferyl, and sinapyl alcohols derived from monolignol biosynthesis, form radicals catalyzed by cell wall laccases and peroxidases. These radicals undergo coupling reactions during the process of lignification to form *p*-hydroxyphenyl (H), guaiacyl (G), and syringyl (S) lignin units, respectively. In addition to monolignol biosynthesis, the flavone tricin [5,7-dihydroxy-2-(4-hydroxy-3,5-dimethoxyphenyl)-4H-chromen-4-one] was recently established as a lignin monomer in grass cell walls in which it potentially functions as a nucleation site for lignin polymerization (del Río et al., 2012; Lan et al., 2015, 2016a). Thus, this discovery linked together two branches of phenylpropanoid metabolism, monolignol and flavonoid biosynthesis, in grass lignification.

Lignocellulosic biomass from *brown midrib* mutants has consistently contained less lignin than their wild-type (WT) counterparts, resulting in forage with increased digestibility for livestock (Porter et al., 1978; Cherney et al., 1991; Vogel and Jung, 2001; Sattler et al., 2010). The *brown midrib* mutants, derived spontaneously or through chemical mutagenesis, have played an important role in identifying a non-redundant set of genes whose products are required in cell wall lignification. In maize and sorghum collectively, five *Brown midrib* (*Bm* in maize; *Bmr* in sorghum) loci have been cloned and characterized, which encode enzymes in either monolignol biosynthesis or *S*-adenosyl methionine (SAM) metabolism. *Bm1* and *Bmr6* encode a cinnamyl alcohol dehydrogenase (CAD) that catalyzes the reduction of cinnamaldehydes to alcohols in the last step of monolignol biosynthesis (Halpin et al., 1998; Saballos et al., 2009; Sattler et al., 2009; Chen W. et al., 2012). *Bm3* and *Bmr12* encode a caffeic acid *O*-methyltransferase (COMT) that catalyzes the methylation of 5-hydroxyguaiacyl groups to form syringyl groups in monolignol biosynthesis (Vignols et al., 1995; Humphreys et al., 1999; Osakabe et al., 1999; Bout and Vermerris, 2003). *Bm5* and *Bmr2* encode a 4-coumarate: coenzyme A ligase that catalyzes the formation of *p*-coumaroyl-CoA, an intermediate in both monolignol and flavonoid biosynthesis (Saballos et al., 2012; Xiong et al., 2019). *Bm2* encodes methylenetetrahydrofolate reductase (MTHFR), and *Bm4* and *Bmr19* encode folylpolyglutamate synthase (FPGS); both enzymes catalyze reactions in SAM synthesis (Tang et al., 2014; Li et al., 2015; Adeyanju et al., 2021). The cofactor SAM is a methyl donor for the two methyltransferases, caffeoyl-CoA *O*-methyltransferase (CCoAOMT) and COMT in monolignol biosynthesis. The genes encoded at four *brown midrib* loci in maize and sorghum, *bm6*, *bmr29*, *bmr30*, and *bmr31* remain to be identified (Ali et al., 2010; Chen Y. et al., 2012; Sattler et al., 2014).

Classical map-based cloning approaches for delineating the genes underlying a given phenotype are potentially informative, but these approaches are low-throughput and time-consuming. Bulked segregant analysis (BSA) provides a simple approach for rapidly identifying molecular markers tightly linked to the causal gene (Michelmore et al., 1991). This approach requires the creation of a segregating population from the progenies; two bulked DNA samples are generated with contrasting phenotypes and further genotyped with molecular markers. The BSA technique has been used to map many important genes in various crop species (Quarrie et al., 1999; Asad et al., 2012; Wang et al., 2012). The development of next-generation sequencing (NGS) technology, with its ease and cost efficiency, has dramatically accelerated the process of identifying causal genes (Hartwig et al., 2012; James et al., 2013; Krothapalli et al., 2013; Nordström et al., 2013). This approach has been successfully used to identify candidate genes for important traits or phenotypes in rice (Tang et al., 2016; Zheng et al., 2016; Wambugu et al., 2018), sorghum (Krothapalli et al., 2013; Jiao et al., 2018), maize (Liu et al., 2012; Haase et al., 2015), and soybean (Kawashima et al., 2016; Song et al., 2017).

In the current study, the gene encoded at the sorghum *Brown midrib 30* (*Bmr30*) locus was identified and characterized using a BSA–NGS approach with the *bmr30* mutant previously isolated by Sattler et al. (2014). *Bmr30* encodes a chalcone isomerase (CHI) and, unlike other *brown midrib* mutants of maize and sorghum, *bmr30* mutants appear to not directly impair monolignol synthesis. Rather, the loss of CHI activity affects the synthesis of the flavonoid tricin, which ultimately results in reduced lignin deposition.

## Materials and Methods

### Germplasm and Genetic Stocks

The *bmr30-1* mutant was isolated and characterized from an ethyl methane sulfonate (EMS)-mutagenized TILLING population of, BTx623, sorghum (*S. bicolor*) (Xin et al., 2008; Sattler et al., 2014). The mutant referred to as *bmr30-2* (PI 678335) was identified using Sorghum Genomics, Gene Discovery Platform at Purdue University^1^ from a sequenced EMS-mutagenized TILLING population of BTx623, and the seed was obtained through GRIN-Global^2^. Near-isogenic lines of *bmr30-1* in two genetic backgrounds (Wheatland and RTx430; Brown et al., 1936; Miller, 1984) were developed through three cycles of backcrossing *bmr30-1* in BTx623 with these two parental lines as recurrent parent and the leaf midrib phenotype as the genetic marker.

### Plants Growth and Care

Seeds were planted in a soil mixture with a 1:2:1:1 ratio of soil, peat moss, vermiculite, and sand and arranged in a randomized complete block design at the University of Nebraska–Lincoln greenhouse facility. Plants were grown under a 12:12 h light:dark cycle and supplemented with high-pressure sodium lighting; greenhouse temperatures were maintained at 29–30°C during the day and 26–27°C at night, respectively. Watering was conducted daily or as needed and Dyna Green All Purpose 12-12-12 fertilizer was applied weekly. Plants were harvested 5–6 weeks after planting for microscopy, RT-qPCR, and protein analyses. Plants grown for stover analyses were grown to maturity.

### Generation of the Mapping Population

The *bmr30-1* mutant was backcrossed to BTx623 for three generations creating a BC_3_F_2_ mapping population. BC_3_F_2_ *bmr30* plants were scored for the midrib phenotype when plants were approximately 0.5 m in height. Digital images were collected to document the leaf midrib phenotype that was continuously monitored throughout the growth of the plants. Individuals whose leaf midribs were not clearly brown (*bmr*) or green (WT) were not included in the study.

Fully expanded leaves from 25 individuals from each phenotypic class were collected for genomic DNA (gDNA) extraction and whole-genome sequencing (WGS). gDNA was extracted from each individual leaf using a cetyl-trimethyl-ammonium bromide-based (CTAB) DNA extraction buffer (Rogers and Bendich, 1985). A total of 300 ng gDNA per individual was pooled to create each *bmr* and WT pool for sequencing. The individual and pooled DNA samples were analyzed by 0.8% agarose gel-electrophoresis to visualize the integrity of the DNA. DNA pools were sonicated on the Covaris LE200 (Covaris, MA, United States) using a protocol designed to achieve a target size of 350 bp. One microgram of total gDNA per pooled sample was used for Nextera Mate Pair library preparation (Illumina, San Diego, CA, United States) and WGS on an Illumina HiSeq X platform (Illumina, San Diego, CA, United States), generating 150-bp paired-end reads. The barcoded libraries were multiplexed and sequenced at Hudson-Alpha Institute for Biotechnology (Huntsville, AL, United States)^3^. Approximately 59.5 and 74.2 Gb of high-quality 150-bp paired-end sequence data was obtained for the *bmr* and WT pools, respectively. The gDNA datasets analyzed for this study are available at NCBI’s Sequence Read Archive under PRJNA736969.

### Variant Calling

Paired-end Illumina reads from each WT and *bmr* pooled sample were aligned to the BTx623 *S. bicolor* reference genome (version 3.1^4^) using Bowtie 2 (v2.3.4.1) mem algorithm with default parameters (Langmead and Salzberg, 2012). Alignment files were converted from sam to bam files and subsequently sorted using SAMtools (v1.8) *view* and *sort* commands, respectively. SAMtools *mpileup* (v1.8) (Li, 2011) was used to output variants using the following parameters: “-B -Q 20 -P Illumina -C50 -uf,” results were piped to BCFtools (v1.7) and variants were called with the *view* command (Li, 2011). Single nucleotide polymorphisms (SNPs) were filtered by the following criteria: (1) coverage ≥5 and ≤100, (2) EMS generated mutations result in G:C to A:T transition mutations, therefore only G to A and C to T single nucleotide changes were retained, and (3) SNPs homozygous and heterozygous for the mutant and WT pools, respectively. The effect of each SNP was annotated using SnpEff (v4.3) based on gene models from the *S. bicolor* reference genome (version 3.1^5^). SNPs with large effects on genes (missense, nonsense, splice site acceptor, and splice site donor) were predicted using SnpEff (Cingolani et al., 2012) and retained as candidate causal mutations.

### Confirmation of Mutation in Mapping Population

Derived cleaved amplified polymorphic sequence (dCAPS) markers were designed to interrogate the causal mutations for both the *bmr30-1* and *bmr30-2*, a G to A transition at position 1252 bp in *bmr30-1* and an insertion of ATGA at position 1029 bp in *bmr30-2* of Sobic.001G035600 based on the genomic sequence from BTx623 *S. bicolor* reference genome v3.1 in Phytozome. PCR primers 5′-GCTGGAGTCCATCATCAGGGAGCACG-3′ (forward) and 5′-CGTGCTCCCTGATGATGGACTCCAGC-3′ (reverse) were used to amplify the *bmr30-1* region and introduce an *Ava*I restriction site in the 209 bp product. PCR primers 5′-GAGAATTGCGTGGCGTTCTG-3′ (forward) and 5′-ACA GGCAGGTAGGGTATAGTACCCA-3′ (reverse) were used to amplify the *bmr30-2* region and introduce an *Nco*I restriction site in the 176 bp product. The *bmr30-1* and *bmr30-2* amplified products were restriction enzyme digested with *Ava*I and *Nco*I, respectively (New England Biolabs) for 2 h at 37°C following the manufacturer’s conditions. The digested samples were analyzed using 4.0% agarose gel-electrophoresis. The *Ava*I restriction enzyme cleaves only the WT PCR product resulting in the 183-bp product, whereas the 209-bp product containing the mutation remained uncleaved. The *Nco*I restriction enzyme cleaves only the WT PCR product resulting in the 149-bp product, whereas the 176-bp product containing the mutation remained uncleaved. Controls included a negative control without DNA template and a heterozygous mix (equal parts homozygous mutant and WT gDNA).

### Allelism Test

Cross-pollinations were made between *bmr30-2* as female parent and *bmr30-1* pollen parent. F_1_ seeds from the complementation test, their parents (*bmr30-1* and *bmr30-2*) and WT (BTx623) were planted in the greenhouse in summer 2021. The plants were visually classified as being *brown midrib* (*bmr*) or WT when the plants were 6-weeks old. Digital images were collected to document the leaf midrib phenotype from the complementation test. DNA analyses were performed to verify the progeny were the result of cross-pollination.

### RT-qPCR

At 6–7 weeks after germination, the fifth leaf from the base and 10 cm of stalk tissue were harvested, immediately flash-frozen in liquid nitrogen, ground using a freezer mill (SPEX SamplePrep) and stored at −80°C. Total RNA was extracted from tissue from four individual plants per genotypic class. Approximately 100 mg of homogenized plant material was added to 1 mL of TriPure Isolation Reagent (Sigma-Aldrich) followed by RNA extraction and purification using the RNA Clean and Concentrator Kit (Zymo Research). RNA was treated with an on-column DNase treatment (Zymo Research). RNA integrity was confirmed using a 1.8% denaturing agarose gel stained with ethidium bromide (EtBr). RNA quantity was determined using a Synergy Microplate (BioTek Instruments). Total RNA (900 ng) was used for cDNA synthesis with the Transcriptor First Strand cDNA Synthesis Kit (Roche Life Science) and RT-qPCR was conducted using SsoAdvanced SYBR Green Supermix (Bio-Rad) using the Bio-Rad CFX Connect Real Time System (Bio-Rad, Inc.). Primers used for CHI (Sobic.001G035600) were 5′-TCAGATCGTTAGTTGGGCGG-3′ (forward) and 5′-CAAACACGACGCACAGACAG-3′ (reverse). The Bio-Rad data were analyzed using the housekeeping gene *α-tubulin* (Sobic.001G1070200.1) for normalization and ΔC_*t*_ values, which were subsequently used for statistical evaluation as described below. No-template and no-reverse transcription controls were included to verify the absence of DNA contamination. Four biological replicates were analyzed for each genotypic class in duplicate.

### Western Blot and Immunodetection

Proteins from *bmr30* and WT plants were isolated from ground leaf and stalk tissue collected from the first set of greenhouse-grown plants. Proteins were extracted using an extraction buffer containing protease inhibitor (Sigma-Aldrich Co., P9599) (Sattler et al., 2009). Protein concentrations were measured using the Pierce 660 nm Protein Assay (Thermo Fisher Scientific). Western blot analysis was conducted as previously described in Sattler et al. (2009). Briefly, the membrane was probed with primary antibody raised against the tomato CHI (polyclonal rabbit) at a 1:5,000 dilution (Kang et al., 2014). Actin content was used as a loading control, and determined using a mouse anti-Actin monoclonal antibody (Sigma-Aldrich Co., A0480) at a 1:20,000 dilution. The secondary antibodies goat anti-rabbit (Sigma-Aldrich Co., A0545) and goat anti-mouse (Actin) IgG + horseradish peroxidase (Sigma-Aldrich Co., A4416) were used at dilutions of 1:8,000 and 1:20,000, respectively. The secondary antibody was detected using chemiluminescence with Amersham ECL western blotting reagent (GE Healthcare). Imaging of chemiluminescence was performed on a Bio-RAD ChemiDoc XRS+ instrument (Bio-RAD).

### Chalcone Isomerase Activity

The *Bmr30* coding region (Sobic.001G035600) was synthesized (GenScript) in expression vector pET-30a (EMD Biosciences) into *Kpn*I and *Xho*I restriction sites. The plasmid was introduced into *Escherichia coli* Rosetta R2 cells for protein expression. Cultures inoculated from a single colony were grown to log phase at 37°C, transferred to 18°C, and induced to produce protein for approximately 18 h following addition of 0.1 mM isopropyl β-D-1-thiogalactopyranoside. Soluble protein was extracted by sonication. The expressed protein contained an N-terminal 6×-his tag and was captured on a nickel resin column and eluted using imidazole. Induction of the expressed protein and protein purification were monitored by SDS-PAGE. The mutant version (Gly191Arg; *bmr30-1*) was introduced through site-directed mutagenesis and purified as described above.

The bottom 10 cm of stalk tissue from 6-week-old greenhouse-grown plants were ground to a fine powder under liquid nitrogen. Cold extraction buffer containing 50 mM potassium phosphate pH 8.0, 1.4 mM 2-mercaptoethanol was added to the ground tissue, and pulse-sonicated (Branson Digital Sonifier) on ice (Robbins and Dixon, 1984). The samples were centrifuged at 18,620× *g* for 15 min and the supernatant was collected for the activity assay.

Chalcone isomerase activity was measured using both purified recombinant protein and plant extracts at 22°C in a 0.2 mL reaction volume containing 50 mM HEPES (pH 7.5) and 50 μM naringenin-chalcone dissolved in ethanol (Jez et al., 2000). The assays with plant extracts included 40 mM sodium cyanide to inhibit chalcone peroxidase activity (Bednar and Hadcock, 1988). Disappearance of the substrate naringenin-chalcone was monitored at 390 nm with a (BioTek Synergy H1) spectrophotometer. The protein content in reactions was determined using the Pierce 660 nm protein assay with bovine serum albumin as a protein standard. Velocity was calculated as a pmol s^–1^ mg^–1^ of protein.

### Anthocyanins and Total Flavonoids

Anthocyanins and total flavonoids were extracted from nutrient-deprived seedlings using methods described in Li et al. (2006) with minor modifications. In brief, seedlings were ground in liquid nitrogen and anthocyanins were extracted with HCl/methanol (1:99 v/v) at three times volume to sample weight for 24 h at 4°C. Samples were centrifuged at 19,000× *g* and supernatants measured at 530 nm. Total flavonoids were determined from ground tissue by extracting in 80% methanol for 24 h at 4°C at the same sample to liquid ratio as above. After centrifugation, 10% AlCl_3_ was added to the supernatant to a final concentration of 1% and total flavonoids measured at 420 nm.

### Histochemical Staining

When plants were 7 weeks old, midribs from the fifth leaf and stalk from the top internode under the peduncle were collected and fixed in 25:75 acetic acid:ethanol overnight and stored in 25:75 dH_2_O:ethanol then embedded in 7% agarose. Leica VT1200s vibratome (Leica Microsystems) was used to make 100 μm transverse sections. Sections were stained for 1–2 m in phloroglucinol-20% HCl. For vanillin-HCl staining, sections were treated for 1–2 min in ethanolic vanillin [10% (w/v)], followed with 1 volume of concentrated HCl. Sections were imaged using an Olympus BX-51 light microscope (Olympus Co.).

### Chemical Analyses of Stover

Harvested stover (stalk and leaf tissue) was dried in forced-air ovens at 50°C and subsequently ground in a Wiley mill fitted with a 2-mm mesh screen (Arthur H. Thomas Co.), followed by grinding on a cyclone mill fitted with a 1-mm mesh screen (UDY Co.). Fiber analysis was performed on ground stover to determine cell wall components using a detergent digestion protocol as described by Vogel et al. (1999). Neutral detergent fiber (NDF), acid detergent fiber (ADF), and acid detergent lignin (ADL) concentrations were determined using the ANKOM 200 fiber analyzer (ANKOM Tech Co.). Relative percentage of cell wall components were calculated using component concentrations extracted on a dry weight basis (Sarath et al., 2007). Stover from four biological replicates was analyzed in duplicate.

Stover from *bmr30-1*, *bmr30-2*, and WT plants was treated for thioacidolysis followed by gas chromatography-mass spectrometry (GC–MS) to determine relative lignin subunit composition [*p*-hydroxyphenyl (H), guaiacyl (G), and syringyl (S) lignin units]. Samples were prepared and analyzed as described in Palmer et al. (2008). Analysis was performed in duplicate on four biological replicates per line.

### Bomb Calorimetry

The energy concentration of ground stover samples (see above) were determined using a Parr 6400 bomb calorimeter (Parr Instrument Co.). Approximately 200 mg of dried, ground stover combined with 600 mg of mineral oil was combusted to estimate calories per gram of dry weight. Energy values were calculated by subtracting the energy released from combustion of the mineral oil alone from the combined mineral oil and stover, which was standardized to the sample weight.

### Nutrient Deprivation of Seedlings

Seeds from WT, *bmr30-1*, and *bmr30-2* were germinated on filter paper moistened with autoclaved purified water (NanoPure Technology) at 26°C for 5 days in the dark. After germination, the seeds were transplanted into trays containing sand moistened with reverse-osmosis (RO)-purified water. The seedlings were grown at 26°C with a 12-h day/night cycle in a growth chamber and watered with RO water as needed. After 3 weeks, the seedlings at three-leaf stage were photographed to document the accumulation of red pigments.

### Nuclear Magnetic Resonance Sample Preparation and Enzyme Lignin Isolation

Samples were prepared for nuclear magnetic resonance (NMR) analysis by essentially following methods previously published (Kim et al., 2008, 2017; Kim and Ralph, 2010; Mansfield et al., 2012; Lu et al., 2013; Landucci et al., 2020).

#### Coarse-Milling and Removal of Extractives

Four biological replicates were obtained for each line, the stems and leaves of WT, *bmr30-1*, and *bmr30-2*, a total of 24 samples. Each vacuum-dried leaf or stem sample was ground using a shaker mill (Retch MM400, 50 mL hardened steel jar, 1 mm × 15 mm hardened steel grinding ball, 30 Hz for 2 min). The fine powder was solvent-extracted with RO water (4 × 40 mL), 80% ethanol (4 × 40 mL), and acetone (3 × 40 mL), cycling through the solvent extractions by suspending the solids in the solvent, sonicating the suspension for 20 min, centrifuging to pellet the solids (4500× *g*, 20 min, 4°C), and decanting the solvent. Following solvent extraction, each sample was dried under high-vacuum (Freezemobile 35EL SP Scientific, 15 mT, 48 h) to provide an extract-free cell-wall powder.

#### Ball-Milling

Each sample (500–600 mg) was then planetary ball-milled (Fritsch Pulverisette 7, 20 mL agate jars, 10 × 10 mL agate grinding balls, 22 grinding cycles at 600 rpm for 10 min, with 10 min rest time between cycles, and reversing direction each cycle). An aliquot (40–50 mg) of this material was put aside for whole-cell-wall NMR analysis (not reported here).

#### Enzyme Lignin Preparation

The remainder of the ball-milled powder was transferred to a 50 mL falcon tube, suspended in 40 mL of sodium acetate buffer (25.5 mM, pH 5.0), and treated with 40 mg of crude cellulases (Cellulysin^®^, CALBIOCHEM^®^). Each sample was incubated at 35°C on a shaker at 250 rpm for 48 h. After incubation, the solids were pelleted by centrifugation (4500× *g*, 20 min, 4°C) and the buffer was decanted. The acetate buffer (40 mL) was replenished, the sample vortexed to resuspend the solids, fresh cellulase (40 mg) was then added, and the solids were incubated on a shaker for 48 h. The sample was again pelleted, spent buffer decanted, and then the pelleted solids were washed with RO water (3 × 40 mL) by suspending the solids, pelleting the solids, and decanting the water. The washed solids were frozen (–20°C) and then freeze-dried (Freezemobile 35EL SP Scientific, 15 mT, 24 h) to yield the enzyme lignin (EL) that comprises essentially all of the (polymeric) lignin in the sample, without fractionation, but also containing some polysaccharide and some protein.

### Nuclear Magnetic Resonance Analysis

Nuclear magnetic resonance experiments were performed on both whole-cell-wall gel and EL samples as previously described (Kim et al., 2008, 2017; Kim and Ralph, 2010; Mansfield et al., 2012; Lu et al., 2013; Landucci et al., 2020). The whole-cell-wall gel samples were prepared by suspending 40–50 mg of sample in 0.6 mL DMSO-d_6_:pyridine-d_5_ (4:1, v/v) and sonicating the samples, with occasional mixing by vortexing, until a uniform gel was formed. The EL samples were prepared by dissolving 10–20 mg of sample in 0.6 mL DMSO-d_6_:pyridine-d_5_ and sonicating them, with occasional vortexing, until the solids dissolved. NMR experiments were performed on a Bruker Biospin (Billerica, MA, United States) AVANCE NEO 700 MHz spectrometer equipped with a 5-mm QCI ^1^H/^31^P/^13^C/^15^N cryoprobe with inverse geometry (proton coils closest to the sample). The central DMSO solvent peak was used as the internal reference (δ_*C*_ 39.5, δ_*H*_ 2.49 ppm). The ^1^H–^13^C correlation experiment was an adiabatic HSQC experiment (Bruker standard pulse sequence “hsqcetgpsisp2.2”; phase-sensitive gradient-edited-2D HSQC using adiabatic pulses for inversion and refocusing) (Kupce and Freeman, 2007). HSQC experiments for the EL and whole-cell-wall samples were carried out using the following parameters: acquired from 11.66 to −0.66 ppm in F2 (^1^H) with 3448 data points (acquisition time, 200 ms) and 215 to −5 ppm in F1 (^13^C) with 618 increments (F1 acquisition time, 8 ms) of 24 scans with a 1 s interscan delay; the d_24_ delay was set to 0.89 ms (1/8J, *J* = 140 Hz). The total acquisition time for a sample was 5 h. After zero-filling to 2k × 1k datapoints, processing used typical matched Gaussian apodization (GB = 0.001, LB = −0.5) in F2 and squared cosine-bell in F1 (without using linear prediction). Volume integration of contours in HSQC plots used TopSpin 4.1.1 Mac software, and no correction factors were used; that is, the data represent volume-integrals only. The aromatic signals composition on a ½ S_2/6_ + G_2_ = 100% basis. The sidechains are reported on an A_α_ + B_α_ = 100% basis, in which A = β-ether, B = phenylcoumaran; C = resinol/tetrahydrofuran peaks were too small to integrate and are not reported.

### Statistical Analysis

Data were analyzed using JMP 12.2.0 (SAS Institute Inc.). Data were tested for normality using the Wilkes–Shapiro test and were log-transformed if the data failed to meet normality. Pairwise comparisons among lines were performed using Tukey’s Honest Significant Differences test at α ≤ 0.05.

## Results

### *Bmr30* Encodes a Chalcone Isomerase

**FIGURE 1:**
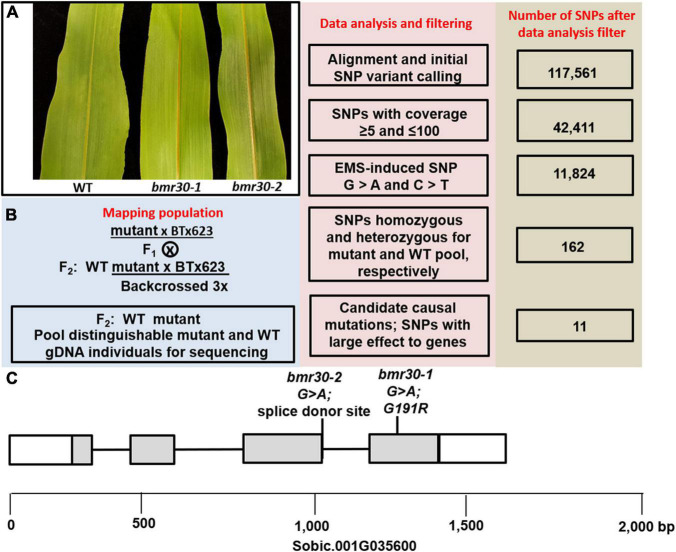
**(A)** The leaf midrib phenotype of the wild-type BTx623 (WT), *bmr30-1*, and *bmr30-2*. **(B)** Overview for gene identification of the mapping population, flowchart of the workflow and number of SNPs from the WT and *bmr30-1* pooled and sequenced gDNA. **(C)** Location of the *bmr30-1* and *bmr30-2* mutations in the *chalcone isomerase* gene, Sobic.001G035600. Boxes and gray shading denote exons and coding sequences, respectively.

A BC_3_F_2_ population segregating for *bmr30-1* was used for a bulked segregate analysis (BSA) and NGS (Figures 1A,B). Pooled gDNA was generated by bulking 25 mutant or 25 WT individuals and was subsequently subjected to high-throughput whole genome resequencing (Illumina HiSeq X platform), which yielded 74 and 59 Gb of 150-bp paired-end data for the mutant and WT pools, respectively. Over 86.8 and 86.0% of the total reads were properly and uniquely mapped to the *S. bicolor* v3.1 reference genome (see text footnote 5), corresponding to an average genome coverage of 80-fold for the mutant and WT pools. Based on alignment to the sorghum reference genome 117,561 SNPs were identified in the mutant and WT pools (Figure 1B). After background mutations were filtered using our data analysis pipeline, 11 homozygous mutations remained for the bulked F_2_ of the *bmr30-1* mutant (Supplementary Table 1). Investigation of the short list with potential candidates contained Sobic.001G035600, which was annotated as a CHI. In *bmr30-1*, a G-to-A transition mutation at position 2,676,019 on chromosome 1 introduced a missense mutation at position 1252 bp in the *CHI* gene, resulting in a substitution of arginine for glycine at amino acid 191 (G191R). To confirm the genetic linkage between the leaf midrib phenotype of *bmr30* and this mutation, a dCAPS marker designed to detect this mutation was used to analyze the individual DNA samples, which were pooled for high-throughput whole-genome resequencing. All the *bmr30-1* individuals were confirmed to be homozygous for the G-to-A mutation (Supplementary Figure 1A). The individuals with the WT midrib phenotype were expected to segregate in a 2:1 ratio (heterozygous:homozygous WT), and 18 heterozygous and 7 homozygous WT for the polymorphism out of 25 individuals (Chi-squared 0.184; *P* > 0.50) were obtained. The *bmr30-1* mutation was introduced into two other sorghum backgrounds, Wheatland and Tx430, through phenotype-based backcross breeding, and DNA marker anlysis confirmed the genetic linkage between this mutation and the leaf brown-midrib phenotype in different lineages (Supplementary Figure 1B).

To corroborate these findings, a second independent mutation was identified in Sobic.001G035600 through an electronic search of the Purdue sorghum TILLING population. A G-to-A mutation at position 1029 bp of Sobic.001G035600, which is the splice donor site (GU) of the third intron (Figure 1C). Seeds from this TILLING line were planted in the greenhouse, and plants with the *brown midrib* phenotype were observed (Figure 1A), which confirms that loss of the Sobic.001G035600 function results in the *brown midrib* leaf phenotype. To determine the impact of the loss of the splice donor site, cDNA was synthesized, and a portion of the cDNA containing the junction between the third and fourth exons was amplified and sequenced from *bmr30-2* plants, which showed loss of the endogenous splice donor site and resulted in an insertion of 4 bp (ATGA) at this junction. This insertion altered the reading frame of the entire fourth exon and changed amino acids 160–231. To confirm the genetic linkage between the leaf midrib phenotype of *bmr30-2* and this mutation, a dCAPS marker designed to detect this mutation was used to analyze an F_2_ population. All the *bmr30-2* individuals were confirmed to be homozygous for the G-to-A mutation at 1091 (Supplementary Figure 2A). To confirm allelism, *bmr30-1* and *bmr30-2* were cross-pollinated, and the leaf midrib phenotype of the F_1_ progeny (*bmr30-2* × *bmr30-1*) were visually assessed when the plants were 6-weeks old. All six plants were determined to have the *brown midrib* phenotype (Supplementary Figure 3).

In the sorghum genome, five genes were previously identified as encoding CHI based on predicted amino acid sequence similarity to the characterized enzyme that converts naringenin chalcone to naringenin in the flavonoid pathway (Nielsen et al., 2016). Phylogenetic analysis of these genes with homologous genes from other flowering plants indicated that *Bmr30* (Sobic.001G035600) resides in a clade with CHIs from other grasses, and the other four sorghum genes were in separate clades (Nielsen et al., 2016). Likewise, the pigments associated with the flavonoid pathway were not completely absent in the *bmr30* mutant tissues, but visible decreases in purple pigmentation were observed in glumes surrounding the seeds (Supplementary Figure 4). To induce flavonoid biosynthesis, the seeds were germinated under nutrient deprivation, which resulted in seedling with red pigmented hypocotyls in WT seedling, but this coloration was visibly reduced in *bmr30-1* and *bmr30-2* seedling (Supplementary Figure 5). Further analysis showed that total flavonoids and total anthocyanin were significantly reduced both by approximately two-fold in *bmr30-2* seedlings relative to WT ones, and a similar trend was observed in *bmr30-1*-seedlings (Supplementary Figures 5A,B). In addition, *bmr30-2* plants had a lesion mimic phenotype, which only became apparent in late vegetative stages (Supplementary Figures 4A,B).

**FIGURE 2:**
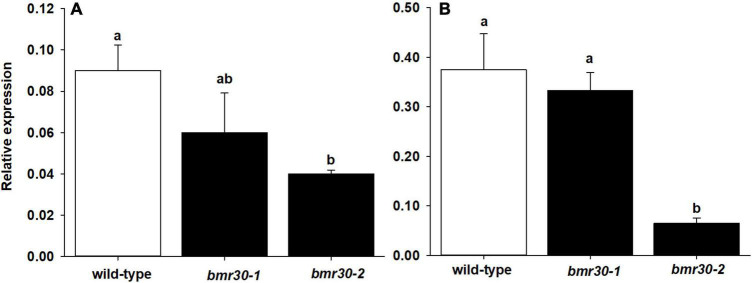
Relative expression levels of *CHI* in **(A)** leaf and **(B)** stalk tissue of wild-type, *bmr30-1*, and *bmr30-2* plants determined using RT-qPCR. Relative expression was determined using the ΔC_*t*_ method with the *α-tubulin* transcript (Sobic.001G1070200.1) for normalization. Error bars represent standard error. Samples with different letters are statistically different at α ≤ 0.05 using Tukey’s HSD test.

The expression levels of *Bmr30* in WT, *bmr30-1*, and *bmr30-2* plants were analyzed by quantitative RT-PCR (Figure 2) from leaf and stalk tissue. *Bmr30* was expressed at low basal levels in WT leaf tissue relative to the control gene *α-tubulin*, and its expression was not significantly different in *bmr30-1* (Figure 2A). *Bmr30* expression in leaf was decreased by 60% in *bmr30-2* compared to WT (Figure 2A). *Bmr30* expression in stalk tissue was not significantly different between WT and *bmr30-1*, whereas expression in *bmr30-2* stalk was significantly decreased, by 83%, compared to WT (Figure 2B). Primers used for RT-PCR were located at the fourth exon just beyond the splice site mutation in *bmr30-2*. Thus, the splice site mutation in *bmr30-2*, which is predicted to cause a loss in function for the gene, appeared to reduce expression of this transcript. The JGI Plant Gene Atlas Project available on Phytozome v13^6^ establishes that expression of CHI (Sobic.001G035600) exists across various tissue types and developmental stages of *S. bicolor* (Supplementary Figure 6). CHI levels in leaves and stalk protein extracts were detected using a polyclonal antibody against a tomato CHI. The levels of CHI were barely detectable and differences among WT and the two *bmr30* alleles were not observed (Supplementary Figure 7), which is consistent with the low expression levels observed by RT-qPCR.

**FIGURE 3:**
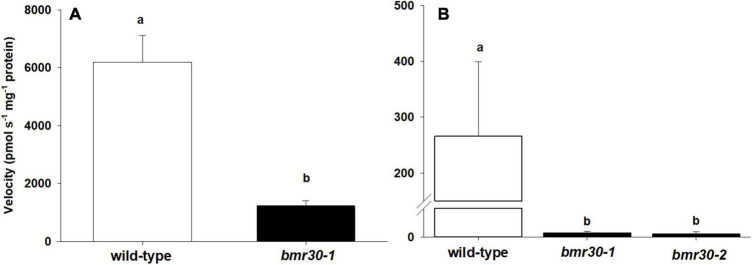
Chalcone isomerase activity from **(A)** purified wild-type recombinant protein and a version containing the *bmr30-1* mutation, and **(B)** plant stalk extracts of wild type, *bmr30-1*, and *bmr30-2*. CHI activity levels were assayed using naringenin chalcone substrate. The enzyme velocity was normalized to the amount of protein (mg) added to the reaction. Error bars represent standard error. Samples with different letters are statistically different at α ≤ 0.05 using Tukey’s HSD test.

The *Bmr30* coding region was cloned, heterologously expressed in *E. coli*, and the protein assayed for CHI activity. In addition, a version containing a G191R amino acid change to replicate the *bmr30-1* allele was also expressed and purified from *E. coli* for a CHI activity assay. Bmr30 catalyzed the conversion of the substrate naringenin chalcone to naringenin. The CHI activity of the G191R version was reduced relative to the WT version of *Bmr30* by five-fold (Figure 3A; *p* > 0.0017). Thus, the product of *bmr30-1* may retain some residual CHI activity. Activity was assayed from crude protein extract from both stalks and leaves. CHI activity were detected in stalk extracts from WT, *bmr30-1*, and *bmr30-2* (Figure 3B), but no activity was detectable in leaf extracts. CHI activity in *bmr30* stalk extracts was reduced approximately 100-fold relative to WT levels (*p* < 0.0001). The undetectable levels of enzyme activity in leaf tissue were consistent with the basal level of gene expression observed with RT-PCR for *Bmr30*.

### Microscopy and Cell Wall Composition

**FIGURE 4:**
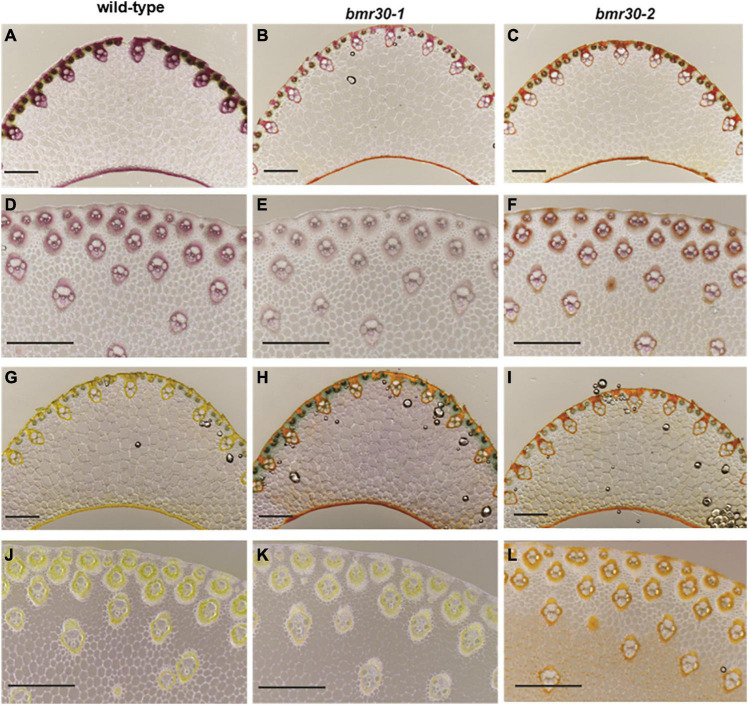
Visualization of lignification using phloroglucinol **(A–F)** and vanillin-HCl **(G–L)** staining of leaf midrib **(A–C,G–I)** and stalk **(D–F,J–L)** tissues from wild-type (BTx623), *bmr30-1*, and *bmr30-2* plants. Scale bar = 500 μm for both leaf midrib and stalk tissue. Leaf midrib tissue was observed at 40× magnification, stalk tissue was observed at 80× magnification using an Olympus BX-51 light microscope (Olympus Co.).

Microscopy following histochemical staining was used to examine how the loss of Bmr30 activity affected the cell walls of leaf midribs and stalks. The cell morphology of both *bmr30* mutants, including fiber cells and xylem of the vascular bundles, closely resembled those cells in WT plants (Figure 4). Secondary cell walls of vascular bundles in both leaf midrib and stalk sections of WT were stained with phloroglucinol-HCl, which reacts primarily with *p*-hydroxycinnamaldehyde end-groups of lignin polymers (Pomar et al., 2002; Figures 4A,D). However, the staining intensity in the vascular bundles of both *bmr30* mutants was reduced compared to WT vascular bundles, which indicated a potential decreased lignin deposition or an alteration in its composition (Figures 4B,C,E,F). Transverse sections were also stained with vanillin-HCl, a staining reagent that reacts with flavonoids (Gardner, 1975). The cell walls of WT leaf midrib and stalk stained yellow in color, which indicated the presence of flavonoids, presumably tricin, within them (Figures 4G,J). In contrast, the cell walls of midribs and stalks from *bmr30* mutants displayed vanillin staining that was considerably different in color and intensity from that in WT, and there were also distinct differences in color and intensity in the staining between the two *bmr30* alleles.

**FIGURE 5:**
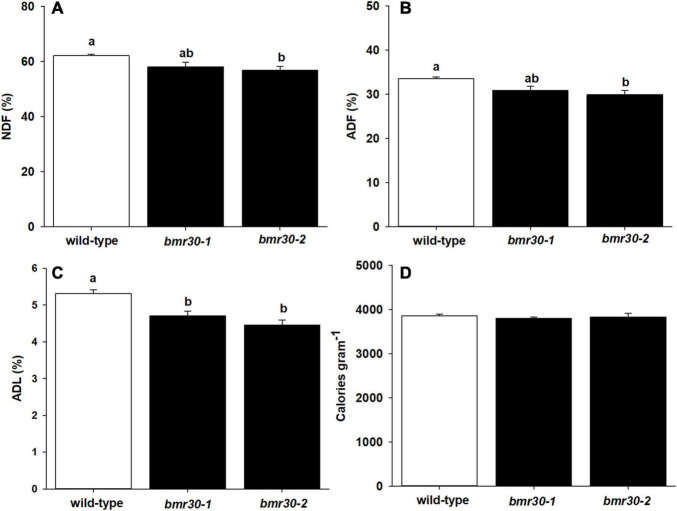
Stover from mature wild-type (BTx623), *bmr30-1*, and *bmr30-2* plants was subjected to fiber analysis to determine percent of **(A)** neutral detergent fiber (NDF), **(B)** acid detergent fiber (ADF), and **(C)** acid detergent lignin (ADL). The NDF, ADF, and ADL were determined using ANKOM fiber analyzer. Stover was also subjected to **(D)** bomb calorimetry to determine total energy using a Parr 6400 bomb calorimeter. Values presented are least squares means (±1 SE). Samples with different letters are statistically different from one another at α ≤ 0.05 using Tukey’s HSD test.

To evaluate how the loss of Bmr30 activity affects biomass composition, fiber analysis was performed on mature stover from WT, *bmr30-1* and *bmr30-2* plants to measure the levels of NDF, ADF, and ADL. Overall NDF, ADF and ADL were all significantly decreased in the *bmr* mutants compared to WT. Specifically, NDF levels were lower in both *bmr30* mutants than in the WT (*p* = 0.0363; Figure 5A). Decreases of 6 and 10% relative to WT were observed in *bmr30-1* and *bmr30-2*, respectively. Similarly, ADF levels were also decreased in *bmr30-1* and *bmr30-2* compared to WT, by 10 and 13%, respectively (*p* = 0.0264; Figure 5B). ADL levels were 11 and 15% lower than WT in *bmr30-1* and *bmr30-2*, respectively (*p* = 0.0024; Figure 5C). The energy density of the biomass was measured using bomb calorimetry. Energy concentrations of stover were not statistically different for either mutant compared to WT (*p* = 0.7909; Figure 5D). The observed reduction in lignin concentration in these *bmr30* mutants is consistent with the previously described effects of *bmr30* on cell wall lignification (Sattler et al., 2014).

**FIGURE 6:**
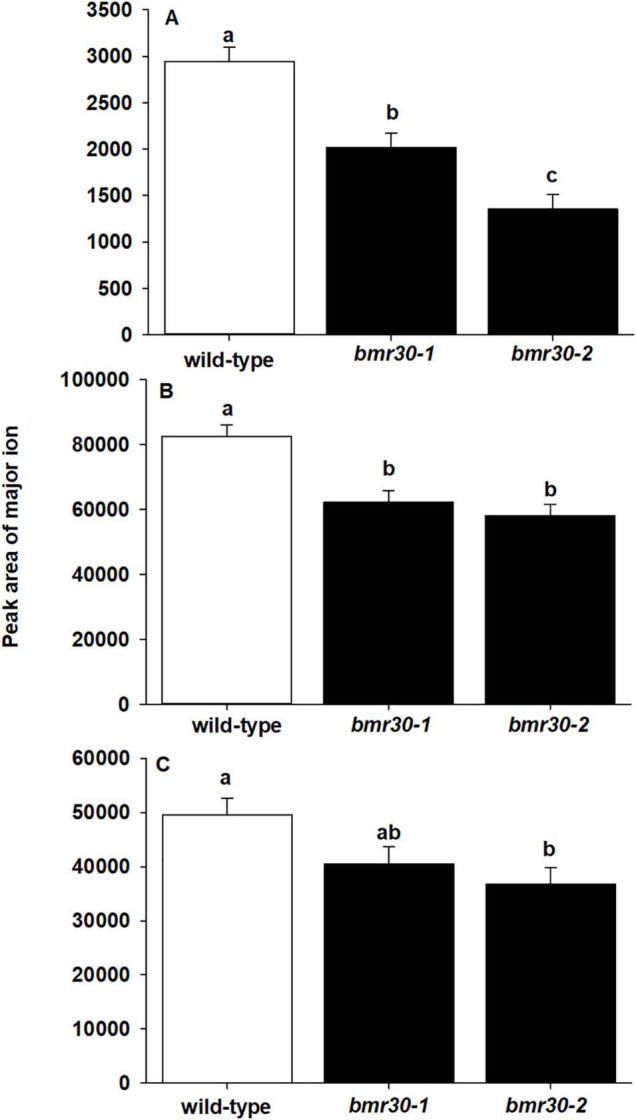
Mutations in *CHI* induced changes in lignin subunit composition. The abundance of **(A)** *p*-hydroxyphenyl (H), **(B)** syringyl (S), and **(C)** guaiacyl (G) lignin subunits from stover of mature wild-type (BTx623), *bmr30-1*, and *bmr30-2* plants was measured using thioacidolysis and gas chromatography–mass spectrometry (GC–MS). Values presented are least square means (±1 SE). Samples with different letters are statistically different from one another at α ≤ 0.05 using Tukey’s HSD test.

Thioacidolysis was performed to determine the composition of β-O-4-linked *p*-hydroxyphenyl (H), syringyl (S), and guaiacyl (G) subunits within the lignin polymer (Figure 6). The levels of H-lignin were significantly decreased in the *bmr30-1* and *bmr30-2* compared to WT (*p* < 0.0001; Figure 6A), with a 31 and 54% decrease, respectively. Levels of G-lignin were significantly different (*p* = 0.0011), with a 25 and 30% decrease in *bmr30-1* and *bmr30-2* compared to WT, respectively (Figure 6B). Similarly, S-lignin was significantly different (*p* = 0.0343), with 18 and 26% decrease in *bmr30-1* and *bmr30-2* relative to WT plants, respectively (Figure 6C). Overall, these two mutations in this *CHI* gene caused a significant decrease in lignin observed through fiber analysis, which was also detected as decreased levels in the three major β-O-4-linked lignin subunits (Figure 5C).

**FIGURE 7:**
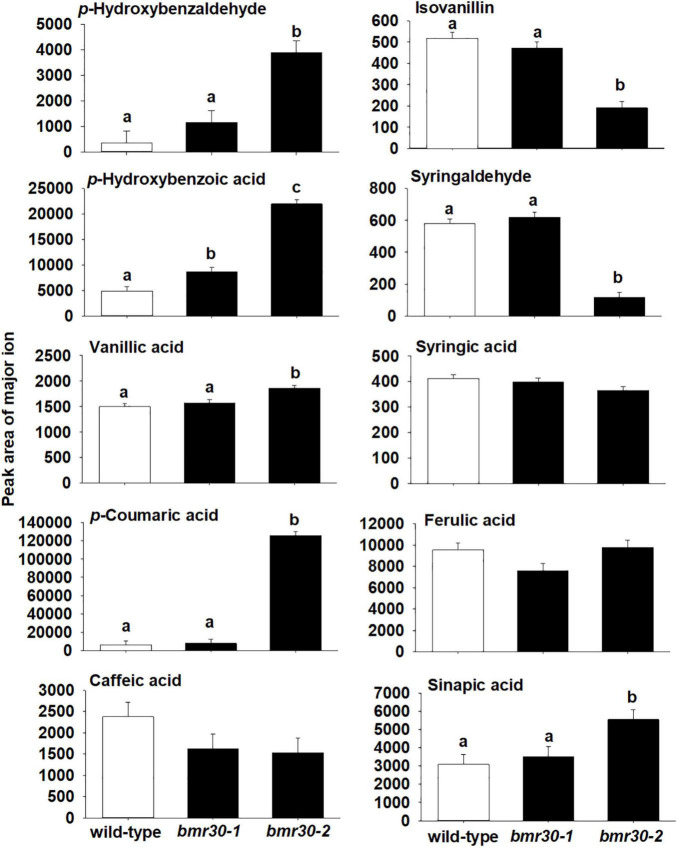
Relative abundance of soluble phenolic compounds in stover from wild-type, *bmr30-1*, and *bmr30-2* plants. Phenolic compounds were analyzed *via* GC–MS. Values presented are least square means; error bars represent standard error. Samples with different letters for soluble fractions are statistically different from one another at α ≤ 0.05 using Tukey’s HSD test.

**FIGURE 8:**
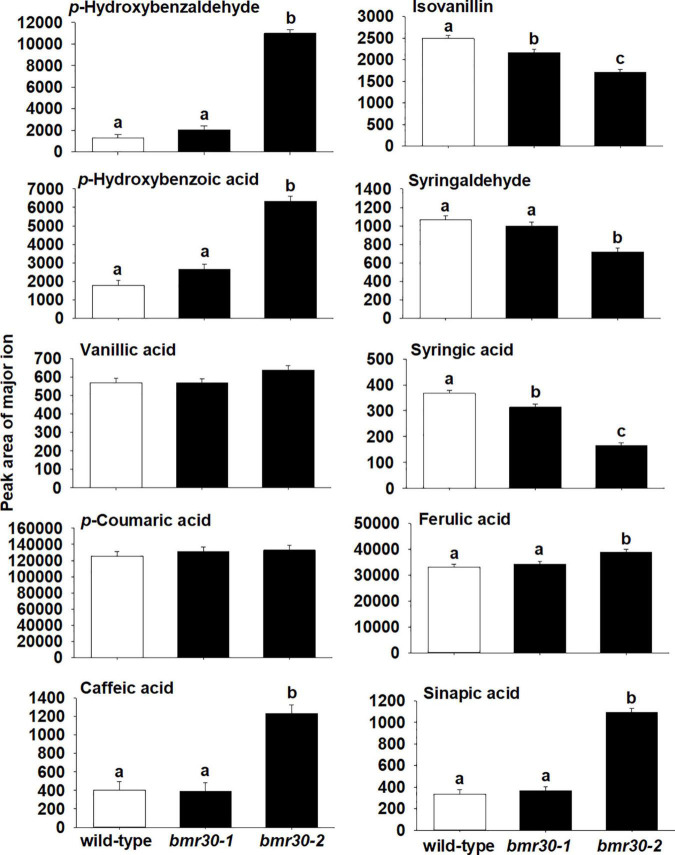
Relative abundance of wall-bound phenolic compounds in stover from wild-type, *bmr30-1*, and *bmr30-2* plants. Phenolic compounds were analyzed *via* GC–MS. Values presented are least square means; error bars represent standard error. Samples with different letters for wall-bound fractions are statistically different from one another at α ≤ 0.05 using Tukey’s HSD test.

To further determine how *bmr30* affects monolignol biosynthesis, soluble and cell-wall-bound phenolic compounds were extracted from stover collected from mature plants. Relative abundances of several phenolic compounds derived from the monolignol pathway were measured by GC–MS (Figures 7, 8). Several soluble phenolic compounds were different among WT and the *bmr30* mutants. For example, *bmr30-2* had significantly higher amounts of *p*-hydroxybenzaldehyde (11-fold greater), *p*-hydroxybenzoic acid (4.5-fold greater), vanillic acid (1.2-fold greater), *p*-coumaric acid (19-fold greater), and sinapic acid (1.7-fold greater) when compared to WT (Figure 8), whereas isovanillin (2.7-fold less) and syringaldehyde (4.9-fold less) were significantly reduced in *bmr30-2* compared to WT (Figure 7). The only soluble phenolic compound that differed between WT and *bmr30-1* was *p*-hydroxybenzoic acid, for which there was 3.3-fold greater amount in *bmr30-1* than in WT. Cell-wall-bound phenolics were also significantly different between WT and *bmr30* mutants (Figure 8). Specifically, *p*-hydroxybenzaldehyde (8.5-fold greater), *p*-hydroxybenzoic (3.6-fold greater), caffeic (3-fold greater), ferulic (2-fold greater), and sinapic (3.3-fold greater) acids were increased in *bmr30-2* compared to WT. Isovanillin (1.3- to 1.5-fold less), syringaldehyde (1.5-fold less), and syringic acid (1.8- to 2.2-fold less) were lower in *bmr30* mutants compared to WT. Taken together, loss of Bmr30 activity affected accumulation of the compounds derived from monolignol biosynthesis.

### Lignin Polymer Analysis

**FIGURE 9:**
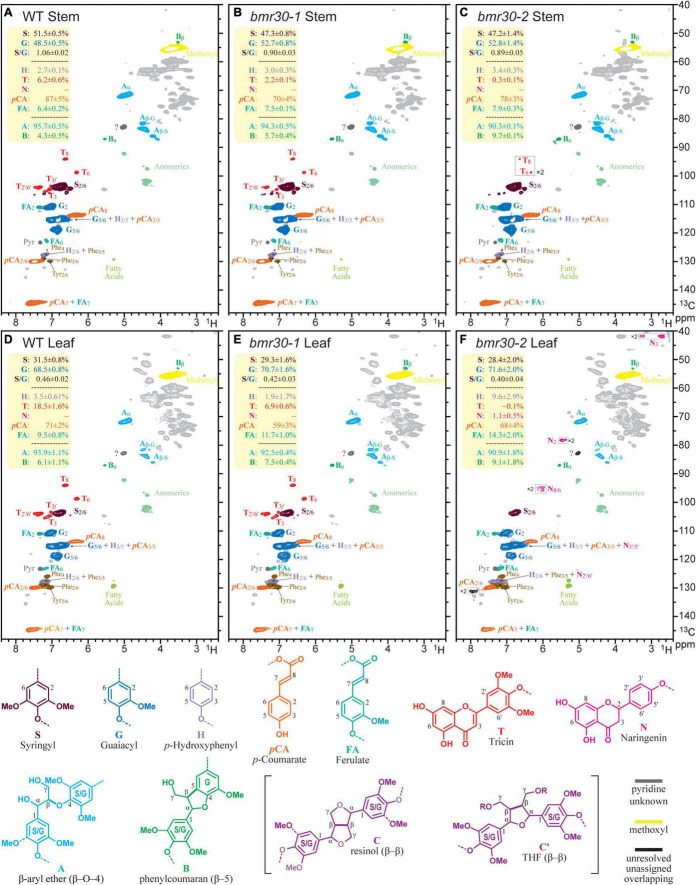
Partial 2D HSQC NMR spectra (in 4:1 DMSO-d_6_/pyridine-d_5_) of the enzyme lignin (EL) preparations isolated from the wild-type and *bmr30* lines. **(A–C)** Spectra of ELs from the stems of wild type (WT), and the *bmr30-1*, and *bmr30-2* mutants. **(D–F)** Analogous spectra from the leaf ELs. Each plotted spectrum is a representative from a set of 4 biological replicates for each; the integral data in the top-left yellow-backgrounded panel of each presents the average and standard deviation from the analysis of all 4 replicates of each line, for a total of 24 samples. The main structural features are, where resolved, colored to match the structures below; no attempt is made to individually color the more minor components in overlapping peaks. The T denotes the tricin peaks, and N the naringenin peaks. Volume integration was used to determine the relative abundances of the G and S lignin units (and the S/G ratio); the other aromatic units including the H, *p*-coumarate (*p*CA), ferulate (FA), tricin (T), and naringenin (N) are reported on an S + G = 100% basis. The H-levels are not reliable because of the overlap with significant phenylalanine (Phe) peaks from proteins, particularly in the leaves, but are corrected by subtracting the volume of the Phe_2/6_ from the volume of the H + Phe_3/5_ peak. Only two of the lignin sidechain structures, characterized by their inter-unit linkages (A, B), were readily determined here, and are expressed as fractions of the sum A + B = 100%. The β-ether content in these samples are not considered to be over 90% of all total units. Resinols C (see structures below) were found at low contour levels (not shown), and the correlation peak labeled? is where the major tetrahydrofuran ${\text{C}}_{\alpha }^{\prime }$ peak occurs. However, the β and γ correlations were not evident (even from TOCSY-HSQC spectra, not shown), so the large peak here is overlapped by another unknown peak; therefore, measurement was not attempted. As previously noted (Mansfield et al., 2012; Abu-Omar et al., 2021), endgroup units such as T and *p*CA, are over-represented in these spectra, but the relative levels remain useful for comparisons.

Changes to the lignin polymeric composition and structure were discerned from NMR profiling (Ralph and Landucci, 2010; Tobimatsu et al., 2019). Extractions for ELs, representing essentially the entire lignin component, were performed by digesting away the majority of the polysaccharides using polysaccharidases (crude cellulase) (Chang et al., 1975). NMR spectra from such lignins are cleaner and sharper than those spectra obtained from whole-cell-wall samples, and were useful here to glean diagnostic details. As shown in Figure 9, the 2D HSQC spectra disperse and resolve various aromatic components in the polymer, including the H, G, and S units in the core lignin, the *p*-coumarates (*p*CA) acylating the lignin sidechain in grasses, the ferulates (FA) that may be on residual arabinoxylans or analogously acylating lignin sidechains, and the flavone tricin (T) that has been found on all Poaceae lignins studied prior to the publication date (Lan et al., 2016b). In the leaf tissue EL of the *bmr30-2* mutant, the intermediary flavanone naringenin (N) was also identified.

HSQC NMR data is not strictly quantitative and the following should be noted: (1) Despite being strictly non-quantitative, the S:G data are considered to be reliable because of the similar environments of the G_2_ and S_2/6_ proton/carbon pairs (Mansfield et al., 2012). (2) The H-level data are distinctly unreliable because of overlap with a significant phenylalanine component (Kim et al., 2017). (3) End-units such as *p*CA and tricin, and perhaps FA, are significantly overestimated due to the longer relaxation of more mobile units than the backbone units in the polymer, but the integrals are useful for comparative analysis (Ralph and Landucci, 2010; Mansfield et al., 2012).

Bearing the above in mind, the following observations are particularly relevant results on the composition and structure of the different lignins. First, the S/G ratio was significantly lower in both the stems and the leaves of the *bmr30* mutants than in the WT, and was essentially equivalent in both mutants. Second, *p*CA levels were similarly lower, which reflected the lower S contents of the mutants and *p*CA units were predominantly (∼90%) on S-units in these samples. Third, ferulate (FA) levels appeared to be higher in the mutants. Fourth, consistent with the loss of CHI activity, the tricin (T) contents were strikingly lower in the mutants, and particularly so in *bmr30-2* (0.1%), than in the WT (18.5%). Naringenin (N) units were detected in the leaf EL from *bmr30-2* (Figure 9E), and perhaps were also detectable in the *bmr30-2* stem EL. This finding is unexpected and appears to be inconsistent with loss of CHI (Sobic.001G035600) function in *bmr30-2*, because naringenin is the product of the CHI enzyme. The incorporation of naringenin, a flavonoid pathway intermediate has been previously documented in a rice flavone synthase (FNSII) mutant (Lam et al., 2017) and most recently in a poplar CHS transgenic (Mahon et al., 2021) and in papyrus (Rencoret et al., 2021).

## Discussion

This study demonstrated that the sorghum *Bmr30* locus encodes a CHI, with loss of function affecting monolignol and flavonoid biosynthesis and lignin deposition. Many genes encoding CHI from angiosperm plant species have been isolated and characterized. The *brown midrib* phenotype, which led to the isolation of the *bmr30-1* mutant (Sattler et al., 2014), has long been linked to C_4_ grasses impaired in cell wall lignification (Jorgenson, 1931; Kuc and Nelson, 1964; Gee et al., 1968; Kuc et al., 1968). Previously identified *bmr*/*bm* loci of monolignol biosynthesis have all encoded enzymes of the monolignol pathway or enzymes involved in the synthesis/recycling of *S*-adenosylmethionine (SAM), a required cofactor for the two methyltransferases of this pathway. However, *Bmr30*, a CHI, breaks this precedent and links two branches of phenylpropanoid metabolism, monolignol and flavonoid biosynthetic pathways, as being required for lignin deposition.

Chalcone synthase (CHS) catalyzes the first committed step in flavonoid biosynthesis that combines *p*-coumaroyl-CoA derived from monolignol biosynthesis and malonyl-CoA from the acetate/malonate-derived polyketide pathway into naringenin-chalcone, which is then isomerized by CHI (CHI) within the cytosol. Bmr30 has CHI activity as demonstrated in this study using versions of the recombinant protein expressed in *E. coli*, and the version G191R to recapitulate the missense mutation *bmr30-1* also retained residual activity, albeit with a 49-fold reduction from WT levels *in vitro* (Figure 3A). The amino acid glycine at position 191 of CHI is highly conserved among *Phaseolus vulgaris*, *Medicago sativa*, *Pisum sativum*, *Zea maize*, *Vitis vinifera*, *Ipomoea purpurea*, *Petunia hybrida*, and *Arabidopsis thaliana* and is two amino acids downstream from residues of the (2S)-naringenin binding cleft (Jez et al., 2000). The substitution of arginine, a charged amino acid with a large side chain for glycine without a sidechain proximal to the substrate binding cleft would most likely impair substrate binding in *bmr30-1*. Likewise, *bmr30-2* contains a 4-bp insertion resulting in a frameshift, which eliminates several secondary structural motifs found in the last 72 amino acids and that includes strands e and f of the third β-sheet and α-helices 6 and 7. α-Helix 6 is highly conserved across all CHIs, and it is part of the active site cleft. Amino acids 188 and 189 of this helix are proposed to confer substrate specificity (Jez et al., 2000). Thus, this allele encodes a protein which is most likely misfolded and rapidly degraded; hence *bmr30-2* is most likely a null (amorphic) allele. The *Bmr30* gene (Sobic.001G035600; previously designated Sb01g003330) transgenically complemented the CHI mutant *transparent testa 5* (*tt5*) restoring anthocyanin pigmentation in Arabidopsis (Liu et al., 2010). Together these results demonstrate that the *bmr30* mutant contains a defective CHI. However, the expression of the gene is relatively low in leaves not experiencing stress, which is consistent with our CHI assays, the RT-qPCR analysis and previously published RT-qPCR analysis (Liu et al., 2010). There is some genetic redundancy that is responsible for the residual flavonoids and anthocyanins present especially *bmr30-2* under stress (Supplementary Figures 4, 5), and one or more of four other CHI-like sequences in the sorghum genome may be responsible. These results suggest that the function of Bmr30 requires its presence at relatively low levels in plant tissue not under stress conditions, which is consistent with the sorghum gene expression atlas for this gene (Supplementary Figure 6). Alternatively, Bmr30 may only be present in a limited subset of cell types within plants, which could also explain the results observed. Our data indicate that a loss or reduction of Bmr30 activity results in reduced lignin deposition and flavonoid biosynthesis. However, accumulation of pigment in *bmr30-1* (designated line 100) in response to peduncle inoculation of the fungal pathogen *Fusarium thapsinum* was not different from WT (Funnell-Harris et al., 2014). The awns surrounding the kernels were one tissue where the intensity of pigmentation was visibly reduced for both *bmr30* mutants compared to WT (Supplementary Figure 4). Similarly, nutrient-deficiency also showed synthesis of total flavonoids and total anthocyanins were decreased in *bmr30* seedlings compared to WT seedlings (Supplementary Figure 5).

Loss of Bmr30 reduced lignin deposition in both mutants and across four lines of experimental evidence. Cytologically, the phloroglucinol staining showed reduced amounts of lignin in *bmr30* cell walls relative to WT. Likewise, chemical analyses showed reduced ADL and decreased levels of major lignin monomers released by thioacidolysis in *bmr30* relative to WT, which together corroborate this reduction. Recent studies of monocotyledonous plant cell walls using NMR have identified the flavone tricin as an endogenous subunit of the lignin polymer in wheat (del Río et al., 2012), maize (Lan et al., 2015), and sorghum (Eudes et al., 2017), and is predicted to be in all species in the Poaceae (Lan et al., 2016b). Tricin links flavonoid metabolism to lignin deposition, and has been shown to be an authentic monomer of lignin, which acts as an initiation site for the lignin chain (del Río et al., 2012; Lan et al., 2015, 2016a). In addition, tricin may have potential health benefits as an antioxidant, anti-aging, anticancer, and cardioprotective compound (Oyama et al., 2009; Zhou and Ibrahim, 2010; Chambers and Valentova, 2015). In the maize CHS *Colorless2* (*C2-Idf*) mutant, tricin levels were significantly reduced in the lignin polymer (Eloy et al., 2017). Indeed, in the current study, NMR profiling also showed alteration to the composition and structure of the different lignins in both *bmr30* mutants relative to WT. The S contents were lower in the mutants, which led to reduced levels of *p*-coumarate and increased levels of ferulate. The tricin (T) contents were strikingly lower in the mutants as anticipated (Figure 9).

Vanillin-HCl stains flavonoid compounds yellow (Gardner, 1975), as was observed in WT leaf midrib and stalk sections. In contrast to WT, staining was greatly reduced in *bmr30-1* tissue sections, which is consistent with the substantial reduction of tricin in lignins determined by NMR profiling. However, the more intense orange-brown vanillin-HCl staining in *bmr30-2* cell walls may suggest increased accumulation of other flavonoids or phenylpropanoids in this mutant. Likewise, there were substantial increases in several soluble and wall-bound phenylpropanoids in *bmr30-2* stover, particularly in mature tissues. Paradoxically, the presence of naringenin, the product of CHI, in lignin from *bmr30-2* mature leaves (Figure 9E) is not consistent with results from the *bmr30-1* mutant and requires further examination. The most plausible explanation is that an additional, unidentified mutation, present in the *bmr30-2* line causes the accumulation in phenylpropanoids described above. The gene discovery platform did not identify any mutations in other flavonoid-related genes for this EMS-generated line, which included the *FNSII* gene (see text footnote 1). Potentially, the lesion-mimic phenotype observed in the *bmr30-2* may be associated with the accumulation of phenylpropanoid compounds as a response to lesion development. The lesion-mimic phenotype is most likely due to a mutation in a gene other than *CHI* (Sobic.001G035600) because the *bmr30-1* mutant does not exhibit this phenotype and neither do *CHI* mutants from other plants (Shirley et al., 1995; Hong et al., 2012; Gurdon et al., 2019). A backcrossing strategy has been initiated to attempt to separate the *brown midrib* phenotype from the lesion-mimic phenotype, which should resolve whether loss of CHI function results in the accumulation of naringenin and hydroxycinnamates in *bmr30-2* leaves and stover.

In summary, the identification of *Bmr30* provides new avenues for the investigation of phenylpropanoid metabolism in sorghum and other C4 grasses. The *bmr30* mutants represent a new class of tools to alter lignin deposition to improve forage for livestock, biofuels, and green chemistry utilization. A future goal will be to combine *bmr30* with other characterized *bmr* mutants that directly impact monolignol biosynthesis to explore the effect on lignin and cell walls on sorghum biomass.

## Data Availability Statement

The datasets presented in this study can be found in online repositories. The names of the repository/repositories and accession number(s) can be found below: https://www.ncbi.nlm.nih.gov/, PRJNA736969.

## Author Contributions

HT and SS designed the research and wrote the first draft of the manuscript. HT, TG, JT, SL, and JR performed the experiments. HT, TG, JT, DF-H, WV, JR, SL, ZX, and SS analyzed and interpreted the data. All authors reviewed and revised the manuscript prior to publication.

## Conflict of Interest

The authors declare that the research was conducted in the absence of any commercial or financial relationships that could be construed as a potential conflict of interest.

## Publisher’s Note

All claims expressed in this article are solely those of the authors and do not necessarily represent those of their affiliated organizations, or those of the publisher, the editors and the reviewers. Any product that may be evaluated in this article, or claim that may be made by its manufacturer, is not guaranteed or endorsed by the publisher.

## Footnotes

1
https://www.purdue.edu/sorghumgenomics/

2
https://npgsweb.ars-grin.gov/gringlobal/search

3
https://gsl.hudsonalpha.org/index

4
https://phytozome.jgi.doe.gov/pz/portal.html

5
https://phytozome-next.jgi.doe.gov/

6
https://phytozome-next.jgi.doe.gov/phytomine

## Supplementary Material

Supplementary Figure 1 The derived cleaved amplified polymorphic sequence (dCAPS) marker for the *bmr30-1* allele was used to verify genetic linkage between *brown midrib* phenotype and the *bmr30-1* mutation from F_2_ individuals that were pooled for next generation DNA sequencing. **(A)** Genomic DNA from 25 individuals with the *brown midrib* phenotype and 25 individuals with the wild-type (green; wt) midribs were screened with dCAPS marker for *bmr30-1*, which was designed to create an *Ava*I restriction site in only the wild-type allele that resulted in a 183 bp product upon restriction digestion. The *bmr30-1* allele lacked this restriction site due to the transition and the 209 bp product was not cleaved by *Ava*I. All individuals with the *brown midrib* phenotype were homozygous for the *bmr30-1* allele, whereas seven individuals were homozygous for the wild-type allele and 18 individuals heterozygous. The latter two groups had normal midribs. **(B)** *bmr30-1* was backcrossed into three different parental lines (Tx623, Wheatland, Tx430) and progeny were phenotyped based on the leaf midrib. These individuals were further tested and confirmed using the dCAPS marker and compared to the original mutant line (*bmr30-1*). PCR amplification controls were performed with DNA from *bmr30-1* (bmr), wild-type (wt), 1:1 mixture of wild-type and *bmr30-1* DNA to simulate a heterozygous plant (het) and no DNA template (-).

Supplementary Figure 2 The derived cleaved amplified polymorphic sequence (dCAPS) marker for the *bmr30-2* allele was used to verify genetic linkage between *brown midrib* phenotype and the *bmr30-2* mutation from F_2_ individuals. Genomic DNA from **(A)** 25 individuals with the *brown midrib* phenotype and **(B)** 25 individuals with the wild-type (green; wt) midribs were screened with dCAPS marker for *bmr30-2*, which was designed to create an *Nco*I restriction site in only the wild-type allele that resulted in a 149 bp product upon restriction digestion. The *bmr30-2* allele lacked this restriction site due to the insertion and the 176 bp product was not cleaved by *Nco*I. All individuals with the *brown midrib* phenotype were homozygous for the *bmr30-2* allele, whereas eight individuals were homozygous for the wild-type allele and 15 individuals heterozygous. The latter two groups had normal midribs. PCR amplification controls were performed with DNA from *bmr30-2*, 1:1 mixture of wild-type and *bmr30-2* DNA to simulate a heterozygous plant (het), wild-type (wt), and no DNA template (-).

Supplementary Figure 3 The leaf midrib phenotype of the wild-type (BTx623), *bmr30-1*, *bmr30-2*, and F_1_ plants form the complementation test *bmr30-2* × *bmr30-1*. The F1 progeny were confirmed to result from cross-pollination using DNA marker analysis. The eighth leaf was photographed from 6-week-old plants.

Supplementary Figure 4 **(A)** Ten week old plants grown in the greenhouse, **(B)** seventh leaf from the bottom of 10 week old *bmr30-2* plant, displaying the disease lesion mimic phenotype, and **(C)** seeds and glumes from wild-type (WT), *bmr30-1*, and *bmr30-2*. The top row is the abaxial side and bottom row is the adaxial side. The grain was harvested from the bottom of the panicles at approximately 40 days after anthesis.

Supplementary Figure 5 Sorghum seedlings were germinated and grown under nutrient-depleted conditions to induce flavonoid accumulation. The absorbance of **(A)** total flavonoids and **(B)** anthocyanins were measured from the **(C)** seedlings. The seedlings from left to right are WT, *bmr30-1*, and *bmr30-2*, photographed 21 days after germination.

Supplementary Figure 6 Expression abundance of chalcone isomerase (*CHI*; Sobic.001G035600), across diverse tissues from *Sorghum bicolor*. Gene expression data was obtained from The JGI Plant Gene Atlas Project available on Phytozome v13 (https://phytozome-next.jgi.doe.gov/phytomine/aspect.do?name=Expression).

Supplementary Figure 7 Immunoblot detection of CHI from leaves (top) and stalks (bottom). Protein extracts from wild-type (WT), *bmr30-1*, and *bmr30-2* were separated by SDS-PAGE, transferred to membrane, and probed with polyclonal antibodies raised against the recombinant tomato CHI protein. The recombinant SbCHI protein (SbCHI) was included as a positive control. Polyclonal antibodies raised against actin protein were used as a loading control.
