# Supplementary figures and images for: The Sorghum (Sorghum bicolor) Brown Midrib 30 Gene Encodes a Chalcone Isomerase Required for Cell Wall Lignification
Source: Front Plant Sci. 2021 Dec 2;12:732307. doi: 10.3389/fpls.2021.732307 (PMC8674566; doi:10.3389/fpls.2021.732307)

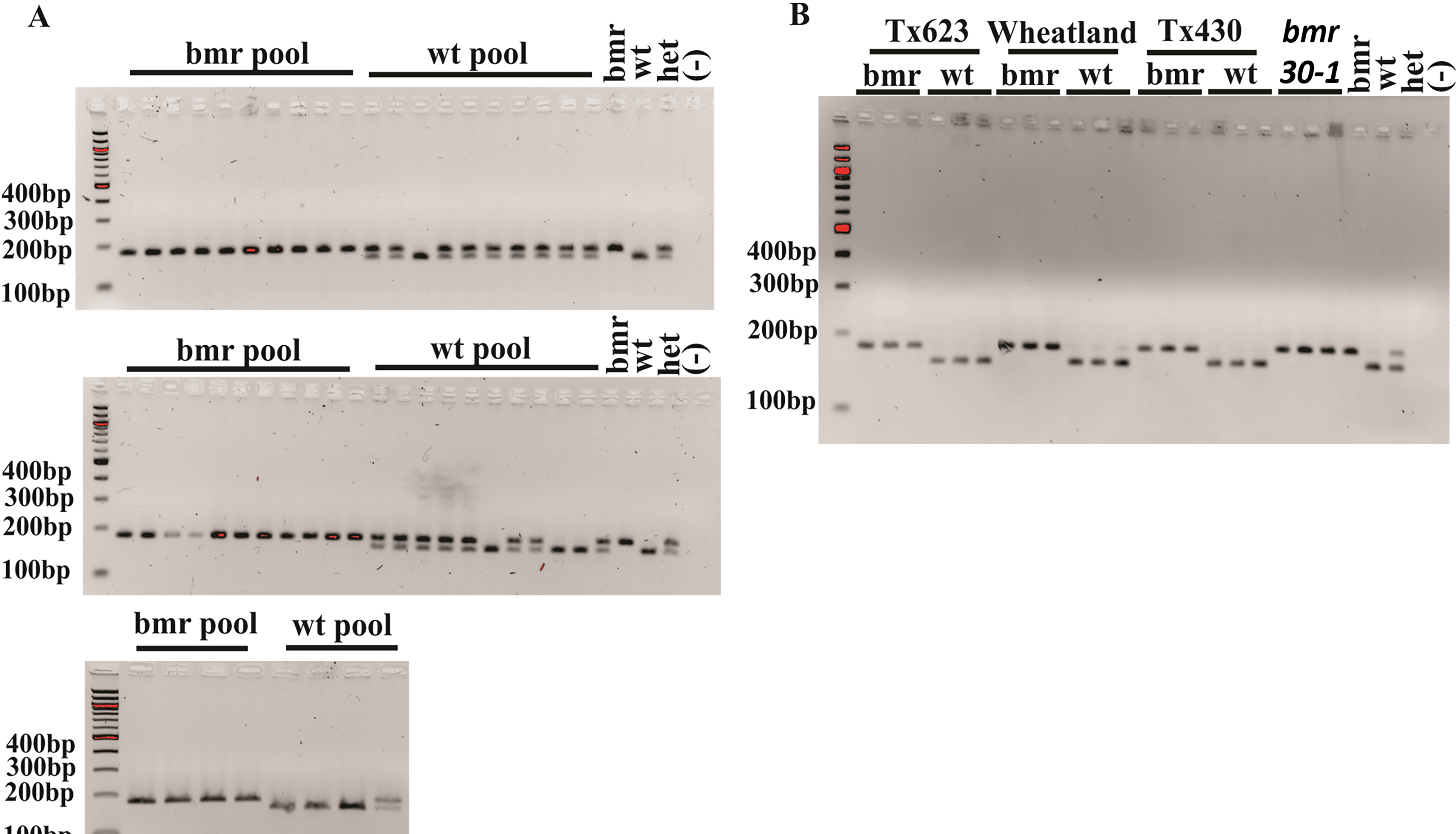

Supplement: Supplementary Figure 1 — The derived cleaved amplified polymorphic sequence (dCAPS) marker for the bmr30-1 allele was used to verify genetic linkage between brown midrib phenotype and the bmr30-1 mutation from F2 individuals that were pooled for next generation DNA sequencing. (A) Genomic DNA from 25 individuals with the brown midrib phenotype and 25 individuals with the wild-type (green; wt) midribs were screened with dCAPS marker for bmr30-1, which was designed to create an AvaI restriction site in only the wild-type allele that resulted in a 183 bp product upon restriction digestion. The bmr30-1 allele lacked this restriction site due to the transition and the 209 bp product was not cleaved by AvaI. All individuals with the brown midrib phenotype were homozygous for the bmr30-1 allele, whereas seven individuals were homozygous for the wild-type allele and 18 individuals heterozygous. The latter two groups had normal midribs. (B) bmr30-1 was backcrossed into three different parental lines (Tx623, Wheatland, Tx430) and progeny were phenotyped based on the leaf midrib. These individuals were further tested and confirmed using the dCAPS marker and compared to the original mutant line (bmr30-1). PCR amplification controls were performed with DNA from bmr30-1 (bmr), wild-type (wt), 1:1 mixture of wild-type and bmr30-1 DNA to simulate a heterozygous plant (het) and no DNA template (-). [file Image_1.TIF]

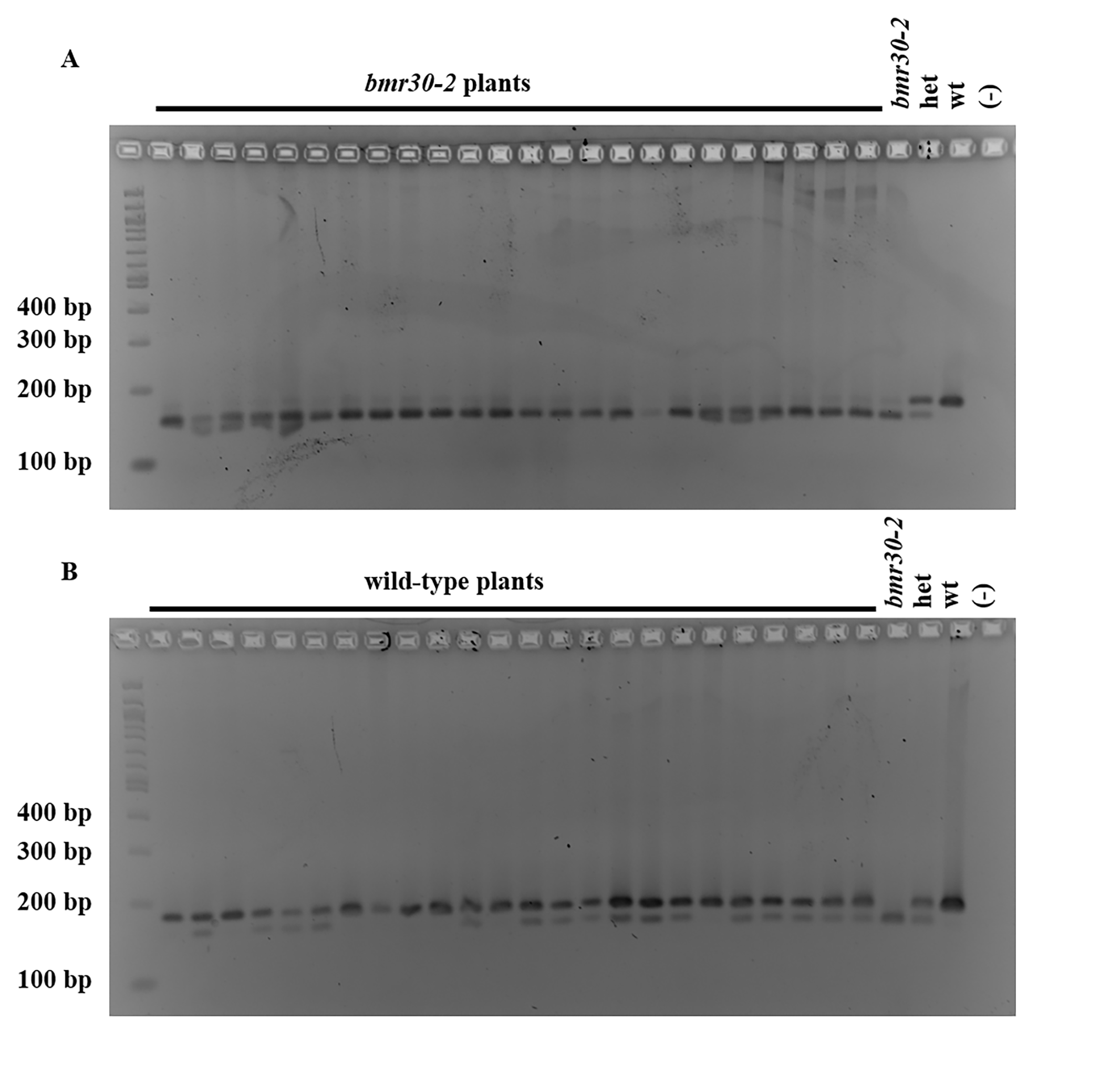

Supplement: Supplementary Figure 2 — The derived cleaved amplified polymorphic sequence (dCAPS) marker for the bmr30-2 allele was used to verify genetic linkage between brown midrib phenotype and the bmr30-2 mutation from F2 individuals. Genomic DNA from (A) 25 individuals with the brown midrib phenotype and (B) 25 individuals with the wild-type (green; wt) midribs were screened with dCAPS marker for bmr30-2, which was designed to create an NcoI restriction site in only the wild-type allele that resulted in a 149 bp product upon restriction digestion. The bmr30-2 allele lacked this restriction site due to the insertion and the 176 bp product was not cleaved by NcoI. All individuals with the brown midrib phenotype were homozygous for the bmr30-2 allele, whereas eight individuals were homozygous for the wild-type allele and 15 individuals heterozygous. The latter two groups had normal midribs. PCR amplification controls were performed with DNA from bmr30-2, 1:1 mixture of wild-type and bmr30-2 DNA to simulate a heterozygous plant (het), wild-type (wt), and no DNA template (-). [file Image_2.TIF]

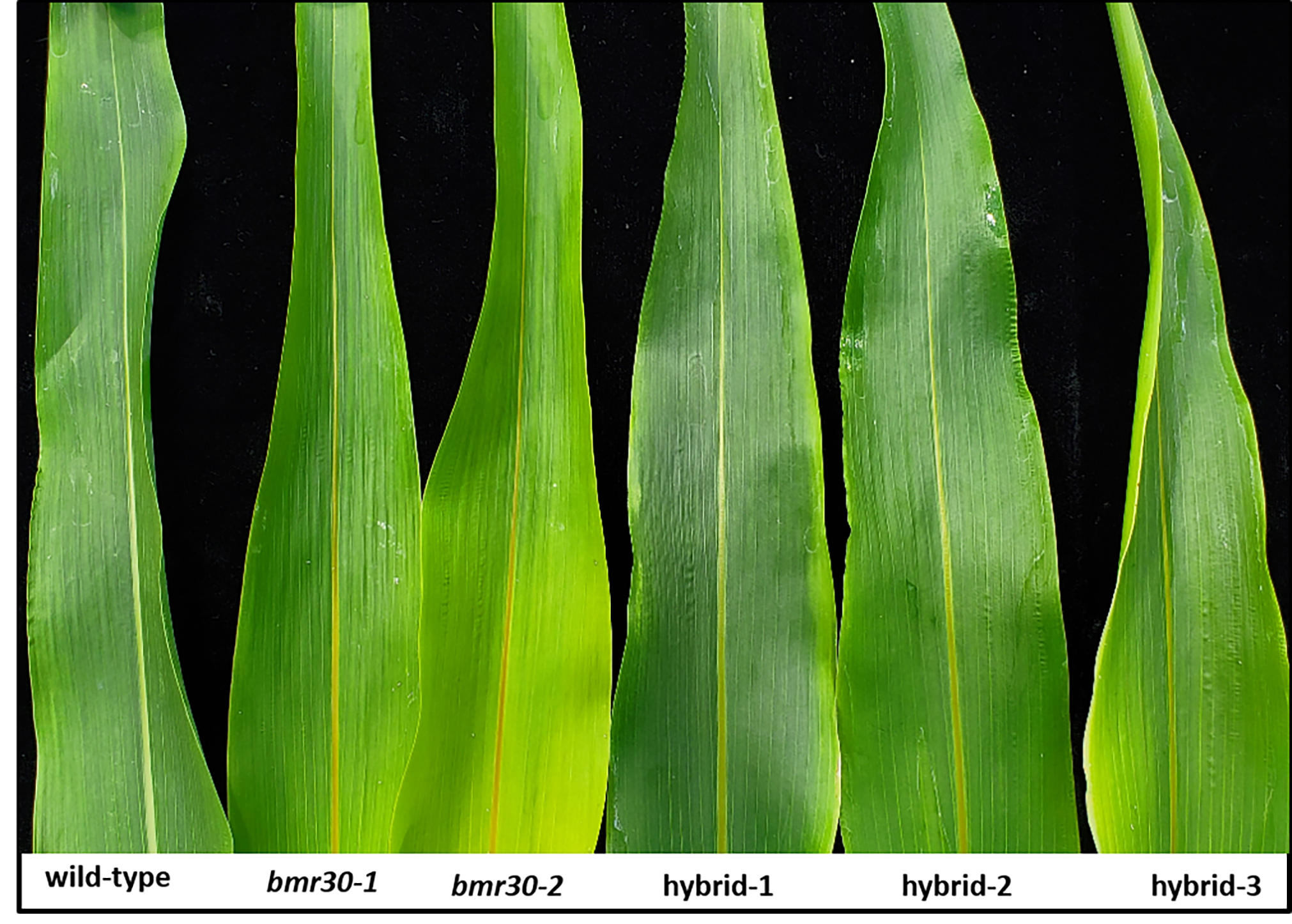

Supplement: Supplementary Figure 3 — The leaf midrib phenotype of the wild-type (BTx623), bmr30-1, bmr30-2, and F1 plants form the complementation test bmr30-2 × bmr30-1. The F1 progeny were confirmed to result from cross-pollination using DNA marker analysis. The eighth leaf was photographed from 6-week-old plants. [file Image_3.TIF]

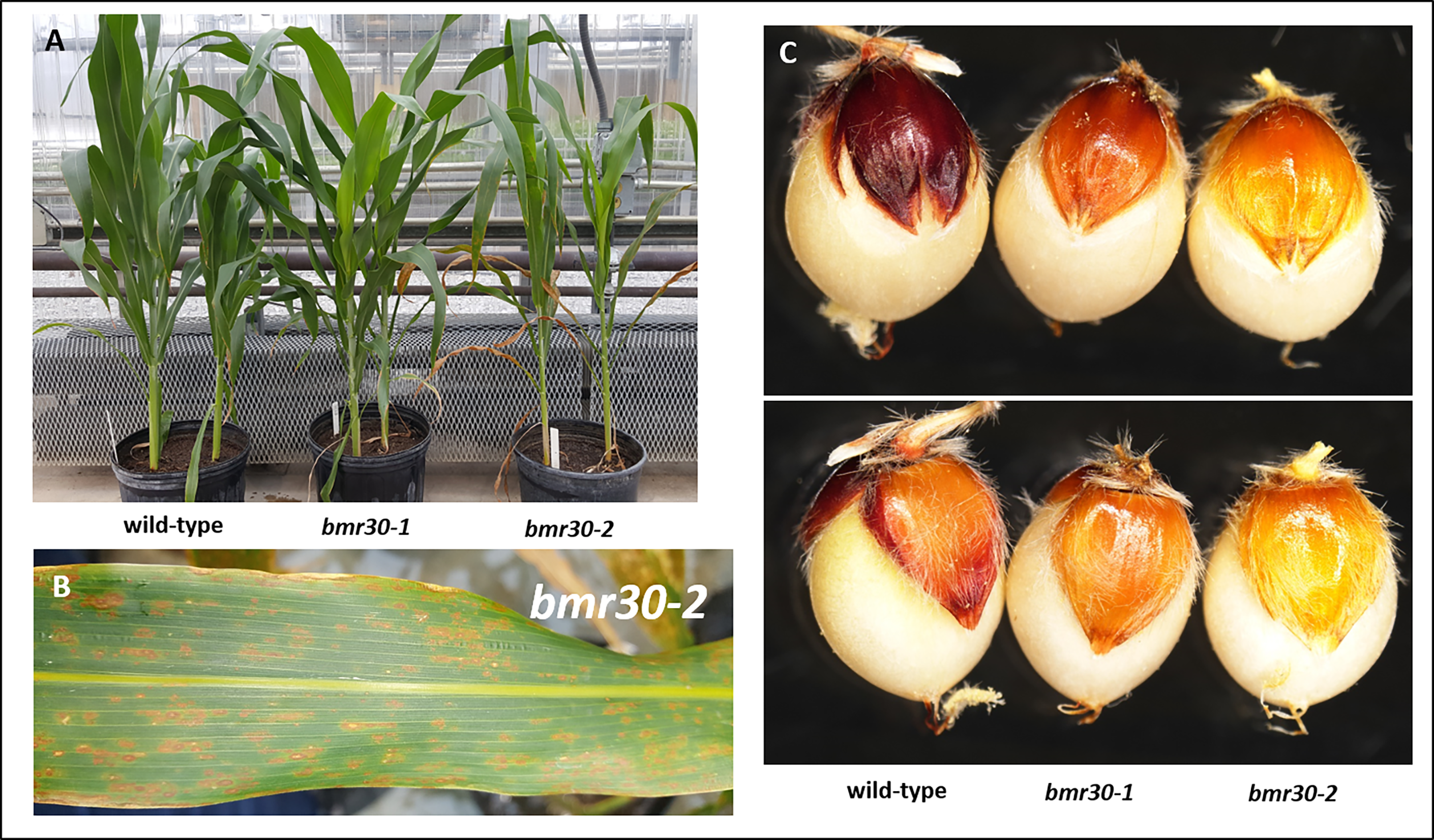

Supplement: Supplementary Figure 4 — (A) Ten week old plants grown in the greenhouse, (B) seventh leaf from the bottom of 10 week old bmr30-2 plant, displaying the disease lesion mimic phenotype, and (C) seeds and glumes from wild-type (WT), bmr30-1, and bmr30-2. The top row is the abaxial side and bottom row is the adaxial side. The grain was harvested from the bottom of the panicles at approximately 40 days after anthesis. [file Image_4.TIF]

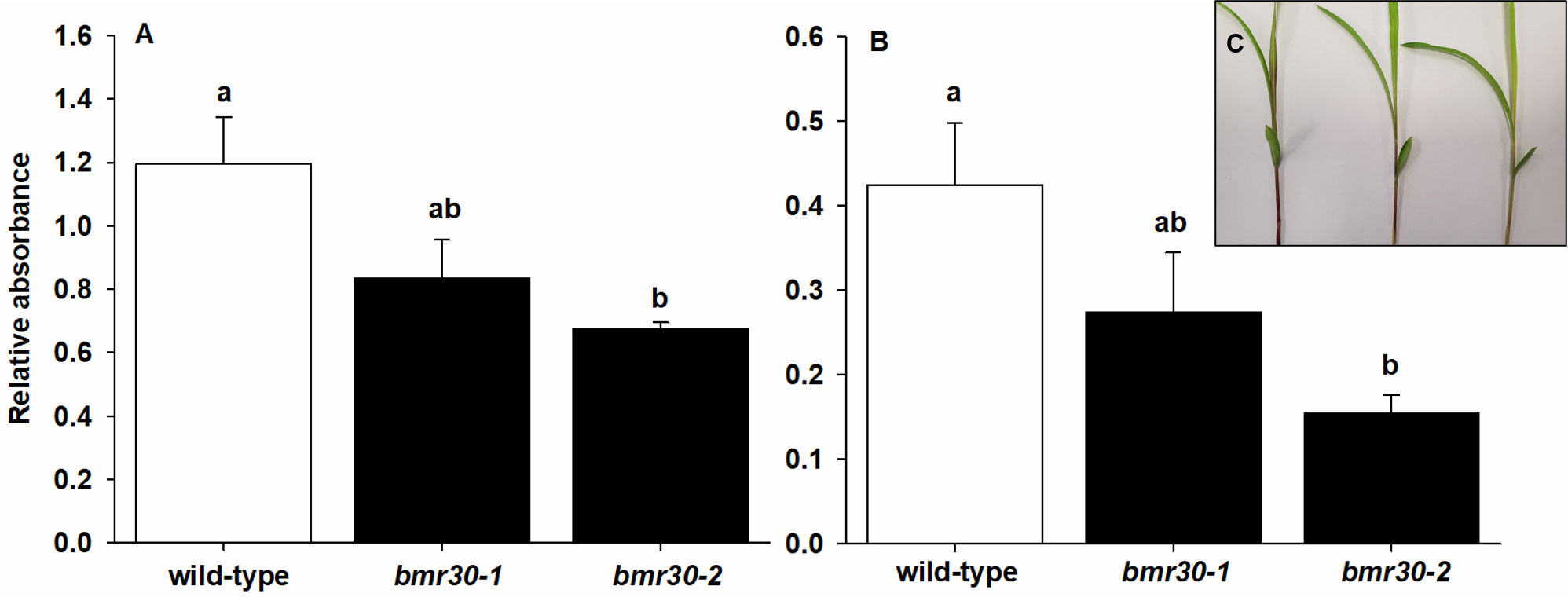

Supplement: Supplementary Figure 5 — Sorghum seedlings were germinated and grown under nutrient-depleted conditions to induce flavonoid accumulation. The absorbance of (A) total flavonoids and (B) anthocyanins were measured from the (C) seedlings. The seedlings from left to right are WT, bmr30-1, and bmr30-2, photographed 21 days after germination. [file Image_5.tif]

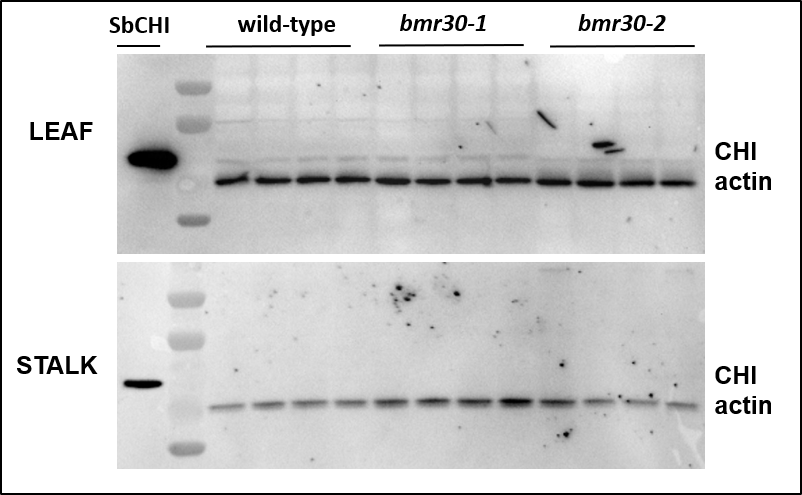

Supplement: Supplementary Figure 7 — Immunoblot detection of CHI from leaves (top) and stalks (bottom). Protein extracts from wild-type (WT), bmr30-1, and bmr30-2 were separated by SDS-PAGE, transferred to membrane, and probed with polyclonal antibodies raised against the recombinant tomato CHI protein. The recombinant SbCHI protein (SbCHI) was included as a positive control. Polyclonal antibodies raised against actin protein were used as a loading control. [file Image_7.tif]
